# Cdk5 activation promotes Cos-7 cells transition towards neuronal-like cells

**DOI:** 10.1515/tnsci-2022-0318

**Published:** 2023-10-25

**Authors:** Li Bao, Xiao-Mei Lan, Guo-Qing Zhang, Xi Bao, Bo Li, Dan-Na Ma, Hong-Yan Luo, Shi-Lu Cao, Shun-Yao Liu, E Jing, Jian-Zhong Zhang, Ya-Li Zheng

**Affiliations:** Department of Pathology, School of Basic Medical Sciences, Ningxia Medical University, Yinchuan 750004, P.R. China; Department of Nephrology, Ningxia Medical University Affiliated People’s Hospital of Autonomous Region of Yinchuan, Yinchuan 750002, P.R. China; Graduate School of Xi’an Jiaotong University, Xi’an 710061, P.R. China; Department of Geriatrics, Ningxia Medical University Affiliated People’s Hospital of Autonomous Region of Yinchuan, Yinchuan 750002, P.R. China

**Keywords:** p35, Cdk5 kinase activity, neuron transformation, NGF

## Abstract

**Objectives:**

Cyclin-dependent kinase 5 (Cdk5) activity is specifically active in neurogenesis, and Cdk5 and neocortical neurons migration related biomarker are expressed in Cos-7 cells. However, the function of Cdk5 on the transformation of immortalized Cos-7 cells into neuronal-like cells is not clear.

**Methods:**

Cdk5 kinase activity was measured by [γ-^32^P] ATP and p81 phosphocellulose pads based method. The expression of neuron liker markers was evaluated by immunofluorescence, real-time PCR, Western blot, and Elisa.

**Results:**

P35 overexpression upregulated Cdk5 kinase activity in Cos-7 cells. p35 mediated Cdk5 expression promoted the generation of nerite-like outgrowth. Compared with the empty vector, p35-induced Cdk5 activation resulted in time-dependent increase in neuron-like marker, including Tau, NF-H, NF-H&M, and TuJ1. Tau-5 and NF-M exhibited increased expression at 48 h while TuJ1 was only detectable after 96 h in p35 expressed Cos-7 cells. Additionally, the neural cell biomarkers exhibited well colocation with p35 proteins. Next-generation RNA sequence showed that p35 overexpression significantly upregulated the level of nerve growth factor (NGF). Gene set enrichment analysis showed significant enrichment of multiple neuron development pathways and increased NGF expression after p35 overexpression.

**Conclusion:**

p35-mediated Cdk5 activation promotes the transformation of immortalized Cos-7 cells into neuronal-like cells by upregulating NGF level.

## 1 Introduction

Cyclin-dependent kinase 5 (Cdk5) is a cyclin-dependent neuronal-specific kinase, which is of great significance in tumor and neurodegenerative disorders [1,2,3]. Although Cdk5 is a member of cyclin-dependent kinase, its kinase activity is activated by p35 and p39 [4,5,6,7]. Cdk5 kinase activity is essential for neurite outgrowth, neuronal migration, and laminar configuration of cerebral cortex and shows highest histone H1 kinase activity in brain [4]. Wu et al. [6] reported that Cdk5 could be detected at embryonic day 12 and continued to increase until postnatal day 7 during neurogenesis, and Cdk5 kinase activity and p35 mRNA exhibited obvious dynamic level in different brain regions and different timepoint of development. Zheng et al. [8] also found that the expression levels of p35 and p39 mRNAs in the marginal zone increase by E15 and E17, paralleling the neurogenetic timetable. Accumulating evidence also reported that p35 and p39 regulated the activity of Cdk5 during neuronal development and function [9,10,11]. Zhang et al. [12] also demonstrated that S-nitrosylation of Cdk5 regulated its kinase activity and dendrite growth during neuronal development. Therefore, p35-mediated Cdk5 activation may play a critical role in neural cell differentiation and development.

Neurodegenerative diseases are characterized by the gradual loss of motor neurons function or death of neuron cells. Except ESCs and iPSCs, dental and oral stem cells, and adult bone marrow and umbilical cord stem cells have been studied for their potential for induction into neuron cells [13,14,15]. Recently, a few studies reported that the disruption of microtubule networks was observed in Cos-7 cells that expressed three isoforms of Tau [3R(−2−3), 4R(−2−3), 4R(+2+3)] with the V337M mutation [16]. Cos-7 is a cell derived from African green monkey kidney fibroblasts and transformed by SV40 virus gene, which showed the expression for neocortical neurons migration related biomarker. Previously, Takahashi et al. [17] demonstrated the generation of iPSC cells from adult human dermal fibroblasts with the four factors: Oct3/4, Sox2, Klf4, and c-Myc, indicating the potential of Cos-7 cells for the induction of neural cells. Li et al. [18] found that the mouse fibroblasts could be directly converted into neuronal cells using only a cocktail of small molecules, with >90% being TUJ1-positive cells after 16 days of induction. This study provided a “proof of principle” for chemically induced reprogramming of fibroblasts. Therefore, Cos-7 cells share some characteristics and potential for the induction of neural cells.

In this study, we investigated the potential of Cos-7 cells for neural cells induction *in vitro* by the artificial expression of p35. Our results showed that p35, as the activator of Cdk5, induced the induction of Cos-7 cells into neuronal-like cells by increasing nerve growth factor (NGF) expression. Cos-7 cells may present us a new option for the sources of neural cells accession in the future.

## 2 Materials and methods

### 2.1 Cell lines and cell culture

Cos-7 cells used in this study was purchased from Institute of Chemistry, Chinese Academy of Sciences (date: 07 May 2021, Shanghai, China; invoice no. 19807823). Re-authentication (STR analysis) of the cell line was performed by the Abace biotechnology, Beijing, on 2 June 2022 (the datasheet is provided in supplementary material). Our study was carried out with mycoplasma-free cells. Cos-7 cells were cultured in RPMI 1640 medium supplemented with 10% heat-inactivated bovine calf serum in a 5% CO_2_ incubator with temperature and humidity set to 37°C and 95%, respectively.

### 2.2 Cell transfection by p35 and Cdk5 expressing vector

Liposome 2000 reagent was used for the transfection of p35, Cdk5, and empty vector (EV) in Cos-7 cells. The transfection medium was replaced with fresh medium after 8 h. Immunofluorescence staining was performed 48 h after p35 transfection.

### 2.3 Cdk5 kinase activity assay

Tissue was homogenized in 150 μL of lysis buffer and sonicated. The lysate was centrifuged at 14,000 *g* for 10 min. 200 μg isolated extract was incubated with Cdk5 antibody overnight at 4℃ with rotating. The immunoprecipitation was washed twice with lysis buffer and twice with kinase buffer. Briefly, 50 μL of kinase assay mixture, 10 μg histone H1, and 10 μL of Cdk5 were used. The phosphorylation reaction was started by 0.1 mM [γ-^32^P] ATP and incubated at 30°C for 30 min. The reaction was stopped by 25 μL of the reaction mixture on p81 phosphocellulose pads. The radioactivity was read in liquid scintillation counter. SDS-PAGE and autoradiography were used to assess the phosphorylated histone H1.

### 2.4 Immunofluorescence

The sterile 20 mm cell slide was placed in 12-well plate, and 1 × 10^5^ Cos-7 cells were seeded in every well. After 8 h transfection and another 48 h culture, cells were fixed in 4% paraformaldehyde at room temperature (RT) for 15 min. The cell slides were permeabilized with 0.5% triton X-100 for 5 min, and blocked with 300 μL of blocking buffer for 1 h at RT. Next 500 μL of primary antibody was added and incubated overnight at 4℃. The slides were washed with PBS three times to remove all unspecific binding of antibody. Then, the cell slides were incubated with secondary antibody for 30 min in darkness. Finally, 200 μL of DAPI (1:1,000) solution was added for 5 min for nuclear staining. The pictures were captured with a laser scanning confocal microscope.

### 2.5 Western blot

The total protein was extracted from 1 × 10^6^ Cos-7 cells. BCA kit was used for protein quantification. 15 μg protein was loaded and run in SDS-PAGE gel. The bands were blocked with 5% skim milk for 1 h at RT. Next the membranes were incubated with the primary antibody diluted in 3% BSA overnight at 4°C. These bands were washed in TBST and incubated with the secondary antibodies for 1 h at RT. Finally, all protein bands were then washed and imaged by chemiluminescence. Grey analysis of bands for agarose gel and western blot were performed by image Lab software. The intensity value was normalized by GAPDH. The primary antibodies were listed as follows: p35/P25: Lot: 2680S, clone number: C64B10, CST; CDK5: Lot: 14145, clone number: D1F7M; CST; Anti-NGF: Lot: ab52918, clone number: EP1320Y, abcam; Anti-Histone H1; #ab4270. The secondary antibodies were listed as follows: Goat anti-mouse IgG secondary antibody: Lot: 3012, Signalway Antibody; Goat anti-Rabbit IgG Secondary Antibody HRP conjugated, Lot: L3012, Signalway Antibody.

### 2.6 Real time-PCR (RT-PCR)

Total RNA was extracted from 1 × 10^6^ Cos-7 cells using TRIzol (Invitrogen) reagent. RNA concentration was measured by NanoDrop 2000 (Thermo). Prime Script RT reagent kit with gDNA Eraser (Takara, RR047Q) was used for cDNA synthesis. Gene expression was detected using the SYBR Premix EX Taq (Takara, RR420A) on an ABI PRISM 7500 (Applied Biosystems). Gene expression was normalized to GAPDH using the 2^−ΔC(t)^ method. The reaction system was as follows: pre-incubation: 95℃, 10 min; amplification: 95℃; 5 s; 60℃, 30 s; 45 cycles; melting curve: 95℃, 5 s; 65℃, 1 min; cooling 40℃, 30 s. The expression levels were quantified using the following primer pairs: GRIN2A-F: 5′-GACCCCAAGAGCCTCATCAC-3′, GRIN2A-R: 5′-CTGGATGGACGCTCCAAACT-3′; GLUR1-F: 5′-TGCTTTGTCGCAACTCACAGA-3′, GLUR1-R: 5′-GGCATAGACTCCTTTGGAGAAC-3′; GLUR2-F: 5′- CATTCAGATGAGACCCGACCT-3′, GLUR2-R: 5′- GGTATGCAAACTTGTCCCATTGA-3′;DLG4-F: 5′-TCGGTGACGACCCATCCAT-3′; DLG4-R: 5′-GCACGTCCACTTCATTTACAAAC-3′; Tau-F: TGAGGACGGATCTGAGGAAC, Tau-R: TGTGGTTCCTTCTGGGATCT; NF-M-F: AGCTGCAGTCCAAGAGCATC, NF-M-R: CAGAGCCATCTTGACGTTGA; GAPDH: F5′-GACATGCCGCCTGGAGAAAC-3′, GAPDH-R: 5′-AGCCCAGGATGCCCTTTAGT-3′. The statistical analysis of gene expression was strictly performed with three technical replicates.

### 2.7 RNA sequence

Cos-7 cells transfected with EV and p35 overexpression vector were collected with 1 mL of TRIzol reagent. RNA was extracted with established protocol. The amplification of all cDNA samples was performed with the KAPA Library Quantification kit for 22–25 cycles. Sequencing libraries were generated by using TruSeq RNA Sample Preparation Kit. AMPure XP system was adopted to purify cDNA fragments of the preferred 200 bp in length DNA fragments. The sequencing library was then sequenced on a NovaSeq platform (Illumina) by Shanghai Personal Biotechnology Co. Ltd. The samples were sequenced to obtain image files, which were converted by the software of the sequencing platform to generate the raw data of FASTQ. We counted the raw data of each sample separately, used Cutadapt to remove the linker at the 3′ end, and removed the Reads with an average quality score lower than Q20. Then, we assessed sequencing data quality by taking base quality distribution, base content distribution, and reads average quality distribution. Finally, the amplified cDNA library was sequenced and analyzed. The fragments per kilobase of transcript per M (FPKM) was calculated and used to estimate the abundance of gene expression.

### 2.8 Elisa assay

The culture supernatant of EV or p35 transfected cells was collected. NGF level was detected according to the manufacture instruments of Elisa kits. Briefly, first, the standard curve was produced and prepared biotin conjugate was added and incubated for 1 h at RT. Streptavidin-HRP solution was added and incubated for 45 min at RT. TMB substrate was added until the substrate began to turn blue. Finally, the Stop Solution was added and the microplate reader was used for quantification.

### 2.9 Statistical analysis

All the experiments were designed for three technical replicates for statistical analysis. Normally distributed data were analyzed by using paired or unpaired two-tailed Student’s *T*-test for single comparisons and two-way ANOVA for multiple comparisons. A *P* value of less than 0.05 was considered as statistically significant.

## 3 Results

### 3.1 p35-mediated Cdk5 activation promoted neurite-like outgrowth in Cos-7 cells

**Figure 1:**
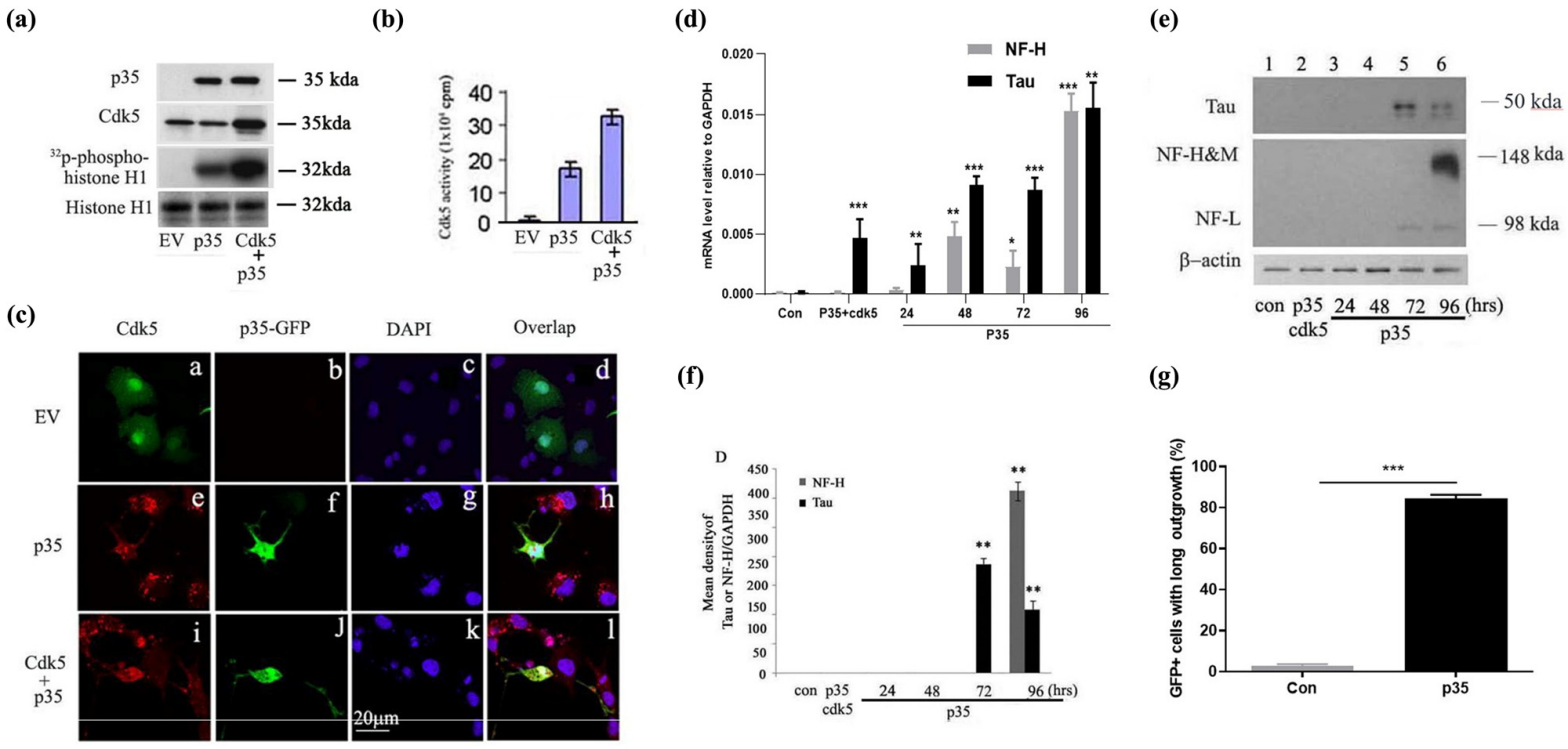
p35 promoted Cdk5 kinase activity and the expression of neuronal-like cell markers in Cos-7 cells. (a) p35 overexpression increased Cdk5 kinase activity. Histone H1 was used as the substrate. and 200 μg total protein was immunoprecipitated by anti-CDK5 antibody. The level of ^32^P phospho-Histone H1 level was analyzed. EV: empty vector; p35: p35 expression in Cos-7 cells; Cdk5 + p35: Cdk5 and p35 co-activation. (b) The quantitative measurement of Cdk5 activity by an *in vitro* kinase assay in Cos-7 cells. (c) Immunofluorescence staining of Cdk5 and p35 protein and the representative graph of neurite-like outgrowth in EV or p35 or Cdk5 + p35 transfected Cos-7 cells. EV: empty vector; p35: p35 expression in Cos-7 cells; Cdk5 + p35: Cdk5 and p35 co-activation. (d) The detection of mRNA level of NF-H and Tau in p35 overexpressed Cos-7 cells by RT-PCR. Con: control. The expression of target genes was normalized to GAPDH. (e) The detected of time-course change of protein level of Tau, NF-H&M, and NF-L by Western Blot in Cos-7 cells ranged from 24 to 96 h after p35 overexpression. (f) The semi-quantitative analysis of Tau or NF-H expression in figure (e). The timepoint ranged from 24 h to 96 h after P35 overexpression. (g) The percentage of GFP^+^ cells with long green tentacles (neuron-like cells) in Cos-7 cells transfected with EV and p35 overexpressed plasmid. *p < 0.05, **p < 0.01. *p* value less than 0.05 was considered to be statistically significant.

To investigate the function of Cdk5 activation in neural cell transformation, we artificially expressed p35 and Cdk5 gene in Cos-7 cells (Figure 1a and b). As expected, P35 and Cdk5 expression exhibited significant increase after p35 and Cdk5 gene overexpression and P35 overexpression did not change Cdk5 expression level in Cos-7 cells (Figure 1a). To further determine the catalytic activity in p35 and Cdk5 co-expressed Cos-7 cells, we performed an *in vitro* kinase activity assay. The Cdk5 protein was extracted by anti-Cdk5 immunoprecipitation. Histone H1 was used as the substrate, and 200 μg total protein was immunoprecipitated by anti-Cdk5 antibody. The results showed that p35 expression significantly upregulated the level of ^32^P phospho-Histone H1 level, which reflected the increased catalytic activity of Cdk5. Moreover, p35 and Cdk5 co-expression Cos-7 cells exhibited higher catalytic activity than cells expressing p35 alone (Figure 1a and b). To investigate the effect of Cdk5 activation on the transformation of Cos-7 cell into neuronal-like cells, we performed immunofluorescence assay of cells transfected with EV, p35 alone, and Cdk5 plus p35. The results showed that p35 was overexpressed in part of cells (Figure 1c, middle panel), which presented higher formation of neurite-like outgrowth than that in p35 non-overexpressed cells (Figure 1c, middle panel). In contrast, Cos-7 cells co-expressing p35 and Cdk5 showed longer green neurite-like tail than that in EV and p35 expressing cells (Figure 1c, low panel, graph I and J). Taken together, p35 mediated Cdk5 activation promoted the formation of neurite-like outgrowth in Cos-7 cells.

### 3.2 p35-mediated Cdk5 activation upregulated the expression of neural cell markers in Cos-7 cells

To further analyze the characteristic of Cos-7 cells with Cdk5 activation, we detected the level of several key neuron cell markers. RT-PCR was performed to quantify the time-course change in Tau and NF-H after Cdk5 activation. The RT-PCR results showed that the expression of NF-H was detectable at 48 h while the expression of NF-H and Tau showed a remarkable increase at 96 h (Figure 1d). Then, we found that p35 plus Cdk5 co-transfection significantly upregulated the protein level of Tau, NF-H&M, and NF-L after 96 h (Figure 1e). The semi-quantification of western blot also showed the obvious increase in Tau and NF-/H/M, respectively at 72 and 96 h (Figure 1f). Besides, we counted the number of neuron-like cells characterized with the cells with long GFP^+^ outgrowth. We found that 85% GFP^+^ Cos-7 cells were neuron-like cells while hardly no neuron-like cells were seen in the population of GFP- cos-7 cells (Figure 1g). Collectively, p35 mediated Cdk5 activation promoted the transformation of Cos-7 cells into neuronal-like cells.

### 3.3 p35-mediated Cdk5 activation contributed to the time-dependent increase neuron cell markers in Cos-7 cells

**Figure 2:**
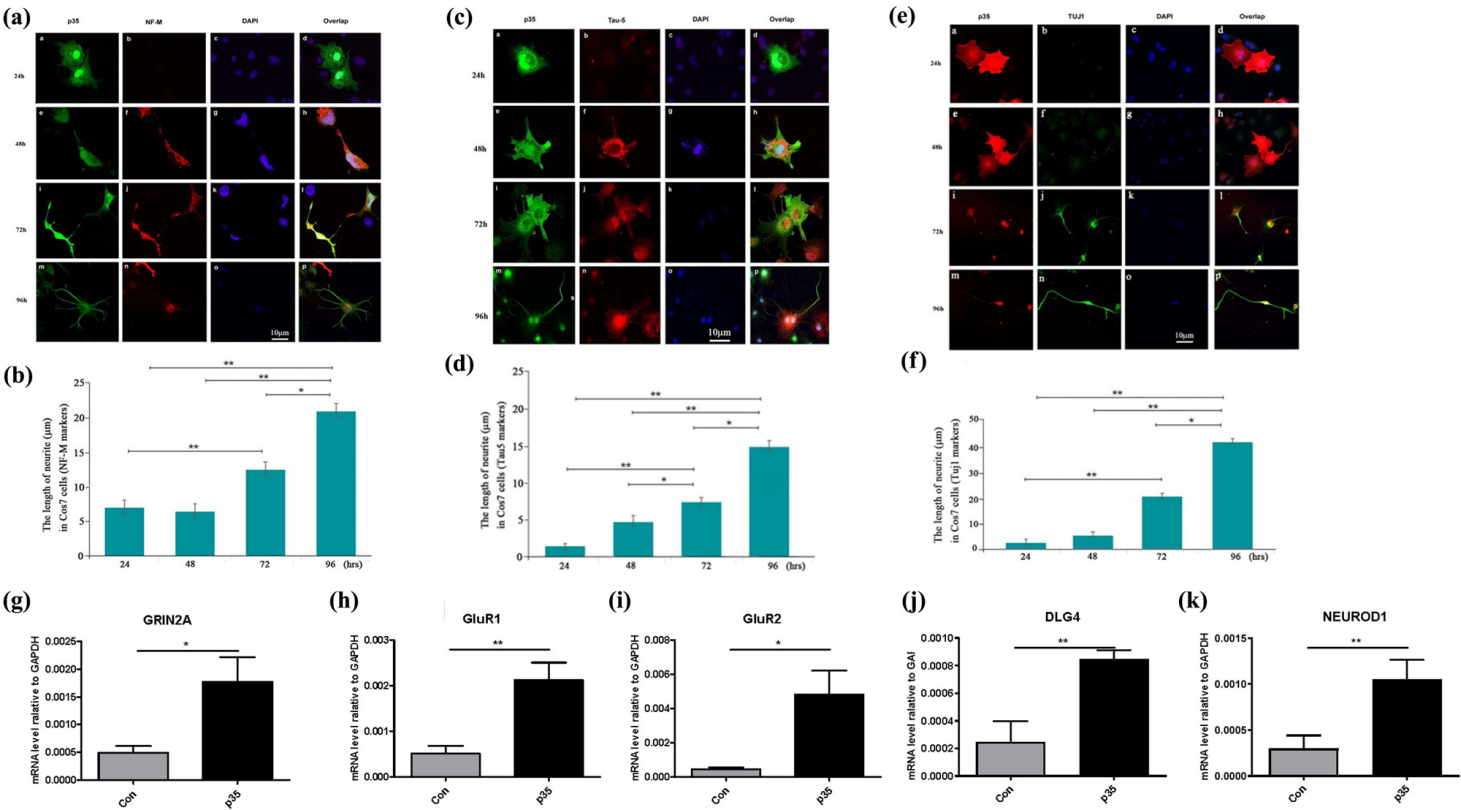
Cdk5/p35 activation upregulates neuron and synaptic related makers level in Cos-7 cells. The (a) representative pictures and the (b) statistics of the length of neurites for the immunofluorescence of NF-M protein at four time points. p35 and NF-M protein were, respectively, marked by green and red fluorescence. The cell nuclei were stained by DAPI. The (c) representative pictures and the (d) statistic of the length of neurites for the immunofluorescence of Tau-5 protein at four time points. p35 and Tau-5 protein were, respectively, marked by green and red fluorescence. The cell nuclei were stained by DAPI. The (e) representative pictures and the (f) statistics of the length of neurites for the immunofluorescence of TUJ1 protein at four time points. p35 and TUJ1 protein were, respectively, marked by green and red fluorescence. The cell nuclei were stained by DAPI. (g)–(j) mRNA level of (g) GRIN2A, (h) GLUR1, (i) GLUR2, (j) DLG4, and (k) NEUROD1 in control and p35 overexpressed Cos-7 cells after 96 h transfection. *p < 0.05, **p < 0.01. *p* value less than 0.05 was considered to be statistically significant.

To clarify the effect of Cdk5 on the change in neural cell markers, we, respectively, recorded the expression of Tau-5, NF-M, and TUJ1 by immunofluorescence at different timepoint after p35 transfection. The results showed that NF-M presented obvious expression after 48 h. Furthermore, we observed that the expression of p35 and NF-M presented high level of co-location on the neurite-like outgrowth (Figure 2a, Graphs h and i). Long neurite-like outgrowth was positively stained by NF-M after 48 h (Figure 2a, Graphs f, j, and n). In addition, we measured the length of NF-M positive neurite and found that NF-M positive outgrowth was statistically elongated after p35 transfection (Figure 2b). We also observed that Tau-5 showed higher expression 48 h after p35 transfection, which was maintained at a stable level in the following time-points (Figure 2c, Graphs f, j, and n; and Figure 2d).

Meanwhile, we also detected the expression of TUJ1, which was a typical molecular marker correlated with neural stem cells differentiation. Compared with NF-M and Tau-5, TUJ1 level was just detectable 72 h after p35 transfection (Figure 2e, Graphs b, f, j, and n) and exhibited the most obvious neurite-like outgrowth after 96 h (Figure 2e, Graphs n and p; and Figure 2f). In addition, to demonstrate the neuronal like characteristics of Cos-7 cells transformed with P35 overexpression, we detected the expression level of several key synaptic and neuron cell marker related genes, including GRIN2A, GLUR1, GLUR2, NEUROD1, and DLG4 by RT-PCR (Figure 2g–k). The results showed that all the five genes exhibited significant increase in p35 overexpressed Cos-7 cells compared with that in EV cells after 96 h (Figure 2g–k). Taken together, p35 mediated Cdk5 expression led to the time-dependent increase in neural cell markers and upregulation of synaptic genes.

### 3.4 p35 overexpression increased NGF expression and resulted in the enrichment of neural differentiation related pathways

**Figure 3:**
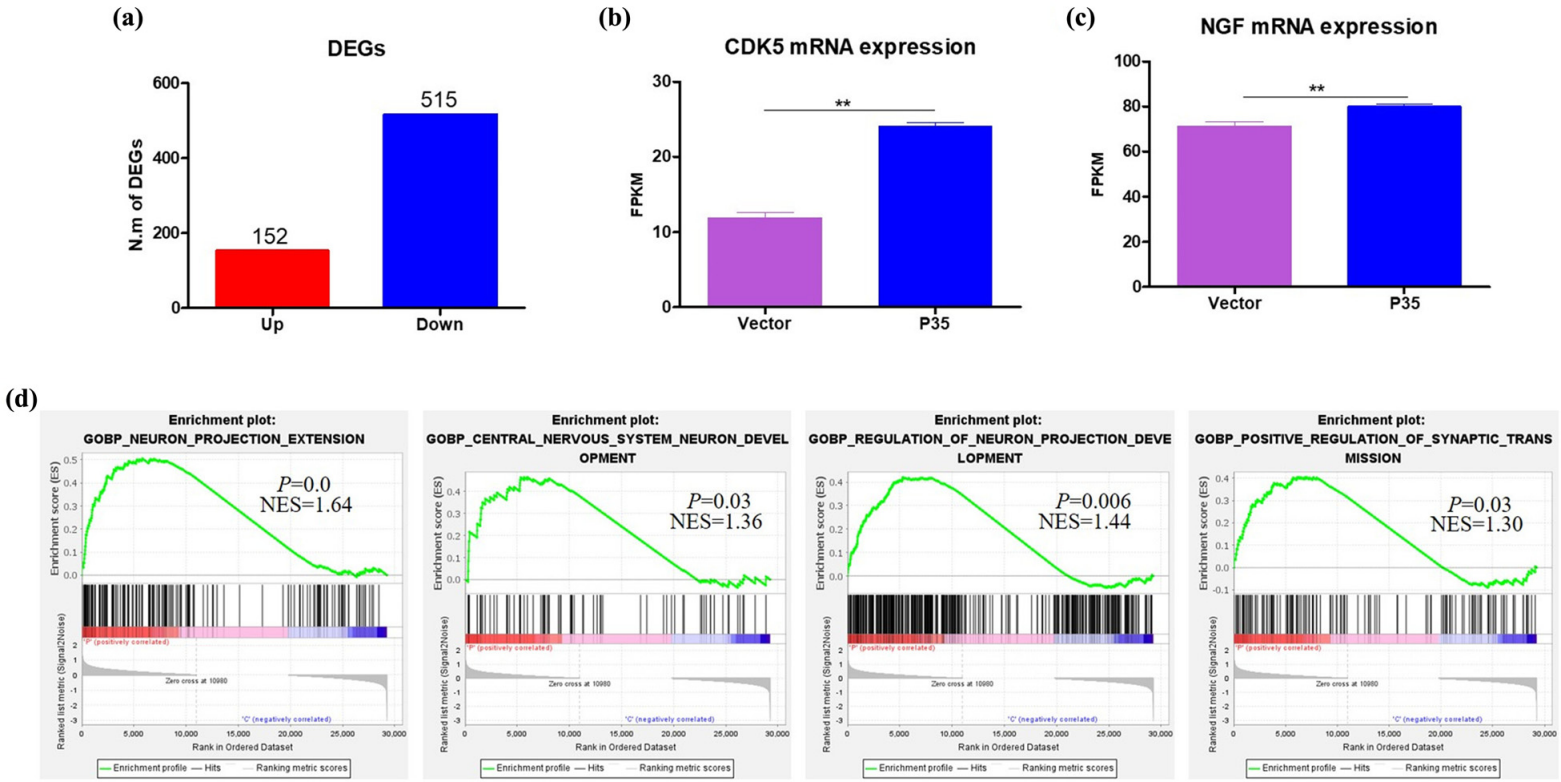
p35 mediated Cdk5 activation increased NGF expression and the enrichment of neural differentiation pathways. (a) The statistics of differentially expressed genes after transfected with empty or p35 vector in Cos-7 cells. The difference threshold was 1.5 times increase or decrease in p35 vector group compared to the EV group. EV, *n* = 4; p35 vector, *n* = 4. (b) The FPKM value of Cdk5 mRNA level after transfected with empty or p35 vector in Cos-7 cells. EV, *n* = 4; p35 vector, *n* = 4. (c) The FPKM value of NGF mRNA level after transfected with empty or p35 vector in Cos-7 cells. EV, *n* = 4; p35 vector, *n* = 4. (d) GSEA of neuron projection extension, nervous system neuron development, regulation of neuron projection development, and positive regulation of synaptic transmission of Cos-7 cells in p35 vector transfection. EV, *n* = 4; p35 vector, *n* = 4.

To investigate the underlying mechanism of p35-induced Cdk5 activation on the transition of Cos-7 cells into neuronal-like cells, we conducted next-generation mRNA sequencing of Cos-7 cells after p35 overexpression. Totally, 667 differentially expressed genes were found between empty and p35 vector group, with 152 genes increased and 515 genes decreased, respectively (Figure 3a). Consistently, we observed the increased mRNA level of Cdk5 in p35 transfected Cos-7 cells (Figure 3b). Importantly, we found that NGF, an important factor for neuron cell development, significantly increased after p35 overexpression in Cos-7 cells (Figure 3c). Gene set enrichment analysis (GSEA) results showed that the neuron development related pathways, including neuron projection extension, nervous system neuron development, regulation of neuron projection development, and positive regulation of synaptic transmission, were significantly enriched in p35 transfected Cos-7 cells (Figure 3d). Therefore, we proposed that p35 overexpression may promote Cos-7 transformation into neuronal-like cell by regulating NGF expression and neuron development related pathways.

### 3.5 p35-mediated Cdk5 activation may promote the transition of Cos-7 cells to neural like cell by the upregulation of NGF

**Figure 4:**
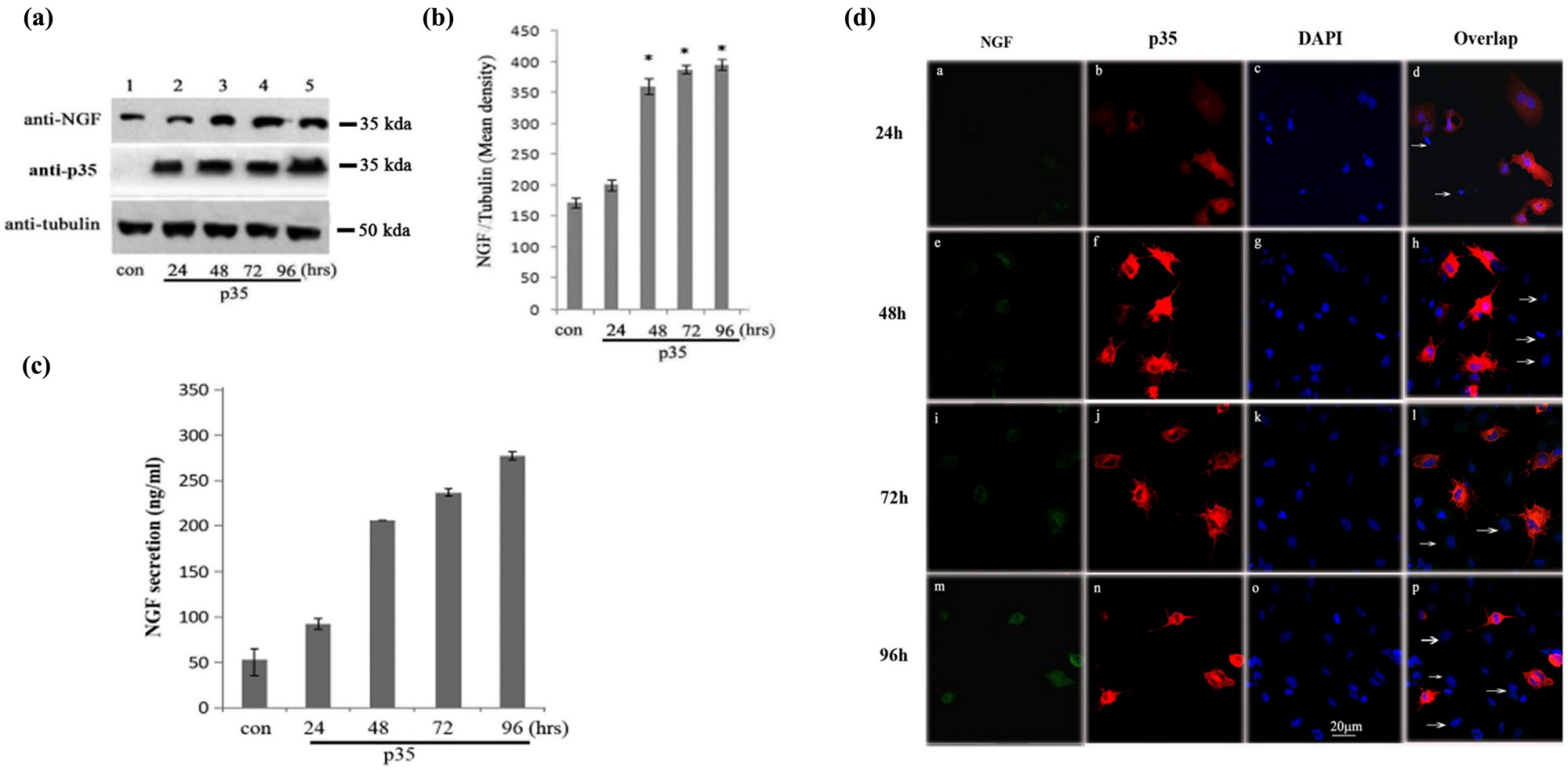
p35 mediated Cdk5 activation increased the expression of NGF in Cos-7 cells. (a) The western blot analysis of NGF time-course expression in Cdk5/p35 activated Cos-7 cells. Four time points, including 24, 48, 72, and 96 h, were set. (b) The statistics of grey analysis of NGF bands in figure (a). (c) The Elisa analysis of NGF levels post Cdk5/p35 activation at different time points in cultural supernatant. (d) Representative graph for the immunofluorescence of NGF protein and four time-points, including 24, 48, 72, and 96 h. p35 and NGF protein were, respectively, marked by red and green and fluorescence. The arrows in Graphs m, n, o, and p represented the p35 negative cells. *p < 0.05. *p* value less than 0.05 was considered to be statistically significant.

To identify the underlying mechanism of p35 expression on Cos-7 cells transformation, we performed further analysis. NGF is the first discovered member of neurotrophic factors, which is discovered for its function on the survival and differentiation of selected populations of peripheral neurons. As mentioned above, p35-mediated Cdk5 activation led to the transformation of Cos-7 cell into neuronal-like cells, we detected the level of NGF after p35 transfection. The western blot assay showed that NGF protein level exhibited significant increase after 48 h and was maintained at a high level in the following time-points (Figure 4a), which was consistent with the expression features of Tau, NF-M, and TUJ1 expression. We also quantified the expression of NGF by calculating the gray value of all the bands, and the result also exhibited the significant increase in NGF in 48 h post p35 transfection (Figure 4b). Then, we performed the Elisa assay to detect the level of NGF in culture supernatant. Consistently, the level of NGF increased significantly 48 h post p35 transfection and reached the highest level at 96 h (Figure 4c). Finally, the immunofluorescence staining showed that p35 transfection led to detectable NGF after 48 h (Figure 4d, Graph e), which presented obvious upregulation after 96 h (Figure 4d, Graphs e, i, and m). Noteworthily, NGF was selectively expressed in p35 positive cells, demonstrating the validity of the specific effect of p35 on NGF expression (Figure 4d, Graphs m, n, o, and p). Collectively, p35 overactivation promotes Cos-7 cell transformation into neuron like cells by the upregulation of NGF.

## 4 Discussion

Previous studies have reported the crucial role of Cdk5 activation in neurogenesis, neurite outgrowth, and neural cell differentiation and apoptosis [19]. In this research, we preliminarily demonstrated that p35-mediated Cdk5 activation leads to the transformation of Cos-7 cells into neuronal-like cells. Furthermore, our results indicated that the upregulation of NGF probably was responsible for the neural transformation of Cos-7 cells after p35 expression.

Cos-7 cells was derived from African green monkey fibroblast cell and was transformed with a mutant strain of SV40. Cos-7 cells mostly resembled human fibroblast cells and, thus, was called Cos-7 fibroblast-like cells. Previous studies demonstrated the transformation of human dermal fibroblasts into iPSCs under the treatment of four stem cell factors, implying the potential ability of Cos-7 cells to differentiate into neuronal-like cells [17]. In addition, a panel of three transcription factors (Myt1I, Brn2, and Ascl1) were able to induce the generation of neurons from mouse fibroblasts. They demonstrated that Ascl1 was the master gene for neuronal fate while the other factors could improve the efficiency of Ascl1 [20]. Recently, Li et al. [18] discovered that the combination of Ascl1, Forskolin, ISX9, CHIR99021, and SB431542 could chemically induce reprogramming of fibroblasts into neuron cells. In addition, miR-124 was also demonstrated to be sufficient to induce trans-differentiation of fibroblasts into functional neurons [21]. In this study, we found p35-mediated Cdk5 activation resulted in the formation of neurite-like outgrowth and the generation of neuronal-like characteristics on the transformed Cos-7 cells. Our results were consistent with the previous research works and testified the ability of Cos-7 fibroblast cell to differentiate into neuronal-like cell by p35-mediated Cdk5 activation.

Cdk5 activation was able to lead to hyperphosphorylated cytoskeleton proteins, such as Tau, neurofilament, and neurofibrillary tangles [22]. Although Cdk5 is expressed in various types of tissues, its activator p35Nck5a is generally considered to be neuron specific [23]. Song et al. [24] reported that interferon γ induced neurite outgrowth by p35-Cdk5-ERK1/2 pathway. In this study, we found that the transformed Cos-7 cells exhibited obvious upregulation of NGF expression, which was an important neuron generation factor. Meanwhile, we also detected the significant increase in NGF in the culture medium after p35-mediated Cdk5 activation. Therefore, our study identified a novel p35-Cdk5-NGF regulating axis affecting the transformation of Cos-7 cells into neuronal-like cells.

The neuronal-like cell induced from Cos-7 cell is a novel source for neuronal-like cell differentiation *in vitro.* Previous studies showed that iPSCs could be induced into CNS neurons and glia [25], and patient-derived iPSCs had been used for modeling and drug screening in Alzheimer’s disease [26,27]. In this study, we investigated the potential of Cos-7 cells in neuronal-like cell differentiation after just p35 overexpression, which is much simple than the protocol for neuronal-like cells induction from iPSCs. In addition, Cos-7 cell is an immortalized cell line, which provided a more accessible and economic source.

Admittedly, our research has several drawbacks. (1) We did not testify the necessity of NGF for the transformation of Cos-7 cells; and (2) the regulating mechanism of Cdk5 on NGF is not clearly clarified.

## 5 Conclusion

We found that p35-mediated Cdk5 activation may transform the Cos-7 fibroblast-like cells into neuronal-like cells by upregulating NGF level. p35-mediated Cdk5 activation may be a promising option for the induction of neuron like cell from Cos-7 cell.
